# Toxic C17-Sphinganine Analogue Mycotoxin, Contaminating Tunisian Mussels, Causes Flaccid Paralysis in Rodents

**DOI:** 10.3390/md11124724

**Published:** 2013-11-28

**Authors:** Riadh Marrouchi, Evelyne Benoit, Jean-Pierre Le Caer, Nawel Belayouni, Hafedh Belghith, Jordi Molgó, Riadh Kharrat

**Affiliations:** 1 Laboratory of Food Toxins, Pasteur Institute of Tunis, University of Tunis Manar, 13 Place Pasteur, Post-Office Box 74, Tunis-Belvédère 1002, Tunisia; E-Mails: riadhmarrouchi@yahoo.com (R.M.); belayouninawel@yahoo.fr (N.B.); 2 Neurobiology and Development Laboratory, Research Unit 3294, National Center for Scientific Research, Research Center of Gif-sur-Yvette 3115, Institute of Neurobiology Alfred Fessard 2118, Gif sur Yvette Cedex 91198, France; E-Mails: benoit@inaf.cnrs-gif.fr (E.B.); jordi.molgo@inaf.cnrs-gif.fr (J.M.); 3 Natural Product Chemistry Institute, National Center for Scientific Research, Research Center of Gif-sur-Yvette 3115, Gif sur Yvette Cedex 91198, France; E-Mail: jean-pierre.lecaer@icsn.cnrs-gif.fr; 4 Analysis Service, Biotechnology Center of Sfax, Post-Office Box K, Sfax 3038, Tunisia; E-Mail: hafeth.belghith@cbs.rnrt.tn

**Keywords:** mycotoxin, toxic Tunisian mussels, liquid chromatography-mass spectrometry, mouse bioassay, neuromuscular system

## Abstract

Severe toxicity was detected in mussels from Bizerte Lagoon (Northern Tunisia) using routine mouse bioassays for detecting diarrheic and paralytic toxins not associated to classical phytoplankton blooming. The atypical toxicity was characterized by rapid mouse death. The aim of the present work was to understand the basis of such toxicity. Bioassay-guided chromatographic separation and mass spectrometry were used to detect and characterize the fraction responsible for mussels’ toxicity. Only a C17-sphinganine analog mycotoxin (C17-SAMT), with a molecular mass of 287.289 Da, was found in contaminated shellfish. The doses of C17-SAMT that were lethal to 50% of mice were 750 and 150 μg/kg following intraperitoneal and intracerebroventricular injections, respectively, and 900 μg/kg following oral administration. The macroscopic general aspect of cultures and the morphological characteristics of the strains isolated from mussels revealed that the toxicity episodes were associated to the presence of marine microfungi (*Fusarium* sp., *Aspergillus* sp. and *Trichoderma* sp.) in contaminated samples. The major *in vivo* effect of C17-SAMT on the mouse neuromuscular system was a dose- and time-dependent decrease of compound muscle action potential amplitude and an increased excitability threshold. *In vitro*, C17-SAMT caused a dose- and time-dependent block of directly- and indirectly-elicited isometric contraction of isolated mouse hemidiaphragms.

## 1. Introduction

Shellfish are exposed to odorous and bioactive secondary metabolites through the ingestion of harmful microalgae, cyanobacteria, consumption of contaminated food and absorption of dissolved compounds from the water column (e.g., phycotoxins, hepatotoxins, cytotoxins, neurotoxins, dermatoxins and odorous metabolites), thus facilitating transfer through the web food chain [1]. Usually, phycotoxins are classified into five categories according to their chemical structures, including derivatives of amino acids (domoic acid), derivatives of purines (saxitoxin and derivatives), cyclic imines (gymnodimines, spirolides, pinnatoxins), non-nitrogenous linear polyethers (okadaic acid, pectenotoxins, azaspiracids) and cyclic polyethers (yessotoxins, brevetoxins, ciguatoxins) [2]. During recent years, an increasing number of toxic events occurred due to emerging toxins as detected by the mouse bioassay without any identification of known toxinogenic organism or toxins [3].

Fungi are known to exist in marine environments [4]. Large number of phytopathogenic and food spoilage fungi (for example, *Aspergillus*, *Penicillium*, *Fusarium* and *Alternaria* species) produce some toxic compounds called mycotoxins [5]. These secondary metabolism products form a polymorphic family with various structures and toxicological properties, including trichothecenes, aflatoxins, sterigmatocystin, fumonisins and *Alternaria alternata* f. sp. *lycopersici* (AAL)-toxins [6,7].

Tunisia has an extensive shellfish industry, especially in Bizerte Lagoon (North) and the Gulf of Gabes (South). Since 1994, incidents involving shellfish contamination have been reported in the Boughrara Lagoon (Southern Tunisia), which obliged the closure of certain production areas. The socio-economic consequences of such incidents were very severe. Later studies revealed that the toxin involved was gymnodimine [8].

In 2006, severe toxicity was detected in mussels from Bizerte Lagoon. This toxicity was demonstrated in most cases using mouse bioassays for detecting diarrheic and paralytic toxins. Analysis of these toxic mussels by liquid chromatography-mass spectrometry (LC-MS), carried out in Ifremer (Nantes, France) and in the Institute for Marine Biosciences (Halifax, NS, Canada), did not identify any conventional phycotoxin profile, suggesting that the toxicity detected in the contaminated mussels was due to new toxin(s).

The aim of the present work was to understand the basis of mussels’ toxicity from shellfish farming areas in Bizerte Lagoon and to characterize through chemical and functional methods the toxic agent implicated in the contamination, and the causative micro-organism(s). The results obtained indicate that the toxic agent is a C17-sphinganine analogue mycotoxin (C17-SAMT). The family of sphinganine-analog mycotoxins (SAMT), which includes fumonisins and AAL-toxins, is named regarding the structural similarity of compounds to sphinganine, the backbone precursor of sphingolipids [9]. A significant account on the chemical and biological characterization of the toxin is provided.

## 2. Results and Discussion

### 2.1. Results

#### 2.1.1. Acute Mouse Toxicity

Mice injected intraperitoneally (i.p.) or intracerebroventricular (i.c.v.) with diethyl ether or dichloromethane mussel extracts, as well as those injected i.p. with only Tween-60 saline, showed neither mortality nor sign of toxicity up to 24 h (*n* = 6 in each case), while an acute toxicity was observed in all mice injected (i.p. or i.c.v.) with the crude water-soluble extract (*n* = 12 in each case). Common mouse toxic symptoms included transient hyperactivity and jumping, followed by flaccid paralysis, and severe dyspnea followed by rapid death (*i.e.*, within 5 min after injection of high amounts of the crude water-soluble toxic extract). The minimum amount of toxic digestive glands which induced mortality of mice was estimated to be 1.25 g/kg (i.p. administration).

#### 2.1.2. Bioassay-Guided Chromatographic Separation

**Figure 1:**
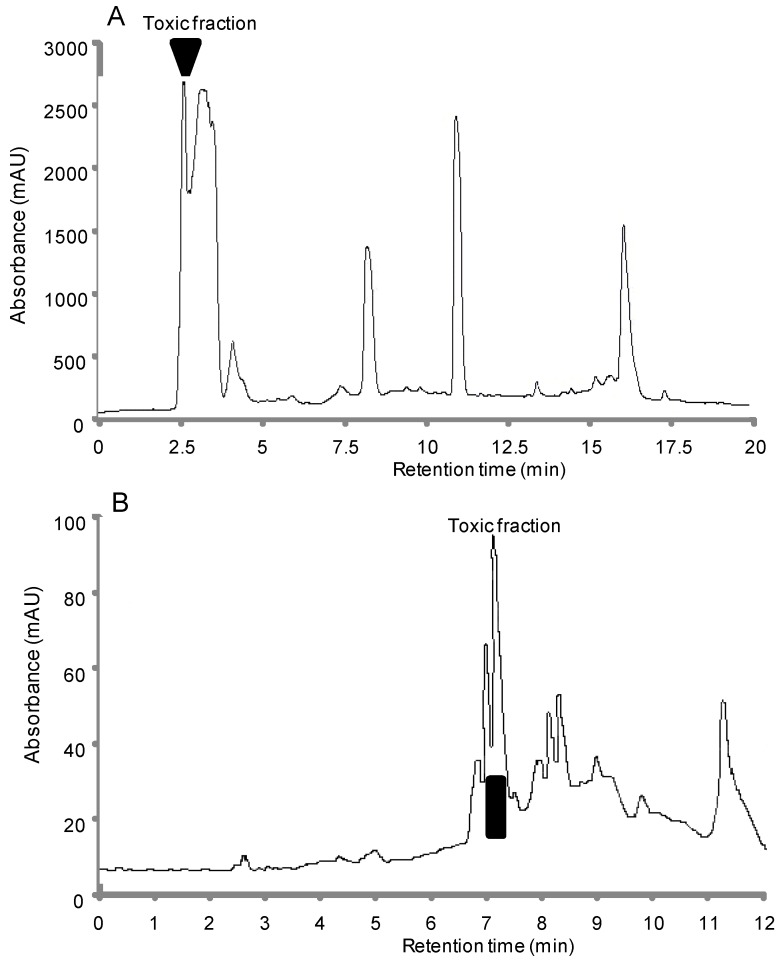
Reversed-phase high-performance liquid chromatography (HPLC) plots of (**A**) the water-soluble neurotoxic extract obtained from mussel samples, using a C18 symmetry column, and (**B**) the water-soluble neurotoxic fraction, possessing the entire toxic activity, using a C18 GOLD ODS column. Elution was performed with a linear gradient of 20%–60% acetonitrile in acidified water run between 2 and 35 min at a flow rate of 1 mL/min. The column effluent was monitored at 210 nm. The active fraction, determined with the mouse bioassay, was repurified under the same conditions to obtain the final purified product.

Eluate fractions from the neurotoxic water-soluble extract, analyzed by reversed-phase high-performance liquid chromatography (HPLC) using a C18 symmetry column, were further assayed for mouse toxicity. The toxic activity was concentrated exclusively in one fraction eluted at the beginning of the gradient (Figure 1A). These results indicated that the toxicity of water-soluble extract was due to this neurotoxic fraction, which was then further purified using a C18 gold octadecyl silica column, under the same conditions, to obtain the final purified toxic product (Figure 1B). A total amount of ~5.2 mg of purified toxic compound was estimated by using LC/MSD Trap XCT system. This compound showed symptoms of toxicity similar to those observed after i.p. and i.c.v. injections of the crude water-soluble toxic extract, and rapid death occurred within 3 min after injections of lethal doses of the purified neurotoxic compound. These paralytic symptoms were similar to those produced by saxitoxin acting on voltage-gated sodium channels [10].

#### 2.1.3. Mass Spectrometry Characterization

**Figure 2:**
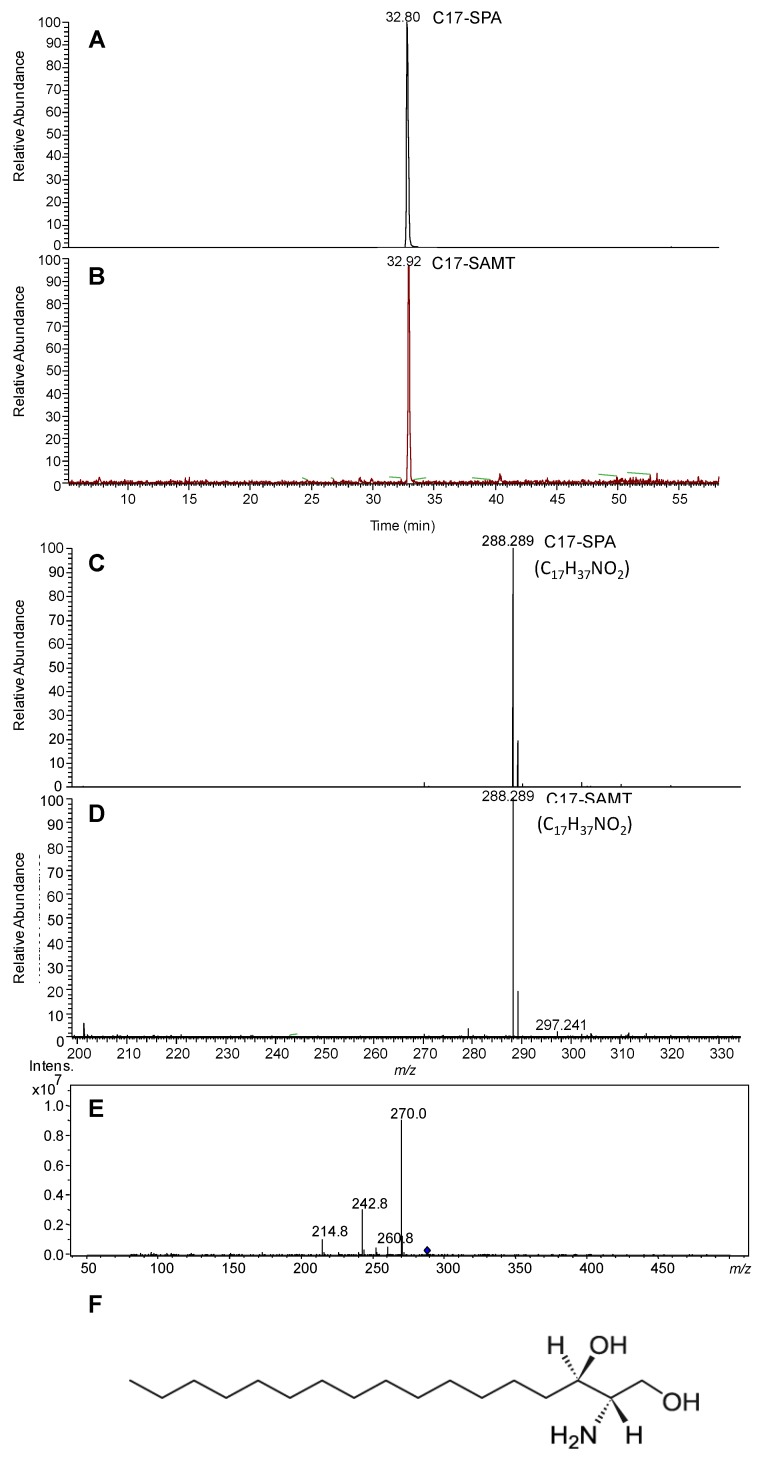
The extract ion chromatogram of the ion 228.289 *m*/*z* and MS spectra (scan range *m*/*z* 200–1800) of C17-SAMT eluted at 32.92 min with a molecular mass of 288.289 (*m*/*z*) (**B**,**D**) and C17-SPA eluted at 32.80 min (**A**,**C**) from LC-MS analysis of water-soluble neurotoxic fraction. (**E**) MS/MS spectrum of selected ion *m*/*z* 288.289. (**F**) Chemical structure of C17-SPA.

The purified neurotoxic compound was eluted at a retention time similar to that of C17-d-erythro-sphinganine (C17-SPA) (Figure 2A,B,F). In addition, the two molecules had an equimolar molecular mass [M + H]^+^ *m*/*z* 288.289 (1 ppm, Figure 2C,D), the theoretical molecular weight of C17-SPA (C_17_H_37_NO_2_) being 287.281 Da. MS/MS spectrum of selected ion 288.289 (*m*/*z*) is shown in Figure 2E. Finally, MS/MS analysis of this peak allowed its unambiguous identification as C17-SPA (462.15 Da) according to its fragmentation profile generating C17-SPA (*i.e.*, 242.8 Da) (Figure 2E). The purified neurotoxic product was named C17-sphinganine analogue mycotoxin (C17-SAMT).

#### 2.1.4. Toxicity of C17-SAMT

The mouse LD_50_ (dose that was lethal to 50% of animals) was 750 and 150 μg/kg (body weight) following i.p. and i.c.v. injections of C17-SAMT, respectively, which was much higher than that of saxitoxin after i.p. administration (mouse LD_50_ = 5–10 μg/kg) [11]. It is worth noting that a mouse LD_50_ of 900 μg/kg was estimated following oral administration of C17-SAMT. Furthermore, a complete recovery of all animals administered with sublethal doses of toxin was observed.

#### 2.1.5. Local *in Vivo* Effects of C17-SAMT

**Figure 3:**
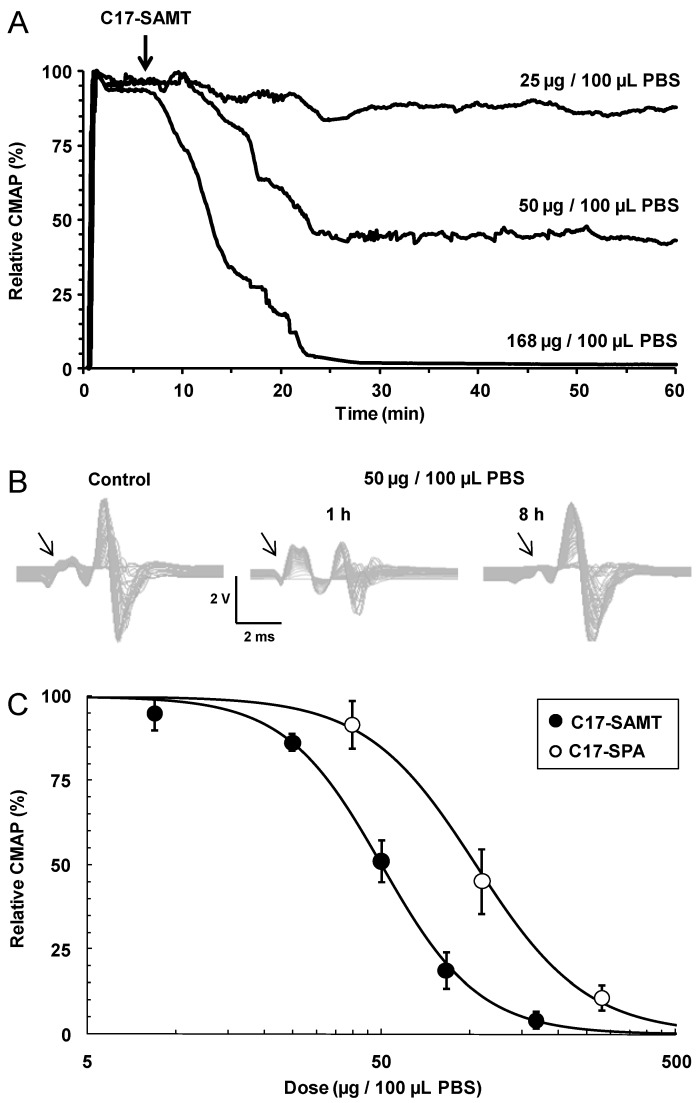
*In vivo* effects of C17-SAMT on the multimodal excitability properties of mouse neuromuscular system. (**A**) On-line recordings of the effect of C17-SAMT (25–168 μg/100 μL PBS) injections on the CMAP maximal amplitude registered continuously as a function of time, from tail muscle following caudal motor nerve stimulation. Values are expressed relatively to those before toxin injections. The arrow indicates the time of injections; (**B**) Traces of CMAPs recorded before and 1 and 8 h after C17-SAMT (50 μg/100 μL PBS) injection. Notice the increased excitability threshold (arrows) after 1 h toxin injection; (**C**) Dose-response curves of the effects of C17-SAMT (black circles) and C17-SPA (white circles) on the CMAP maximal amplitude. Values represent means ± SD of data obtained from 3 to 4 mice, and are expressed relatively to those obtained before toxin injections. The curves were calculated from typical sigmoid non-linear regression through data points (*r*^2^ ≥ 0.996). The toxin dose required to block 50% of the CMAP amplitude (IC_50_) was 50 μg (C17-SAMT) and 108 μg (C17-SPA)/100 μL PBS.

The multimodal excitability properties of the mouse neuromuscular system were studied, *in vivo*, to provide an insight into the mode of action of C17-SAMT. Immediately after the injection of 100 μL phosphate buffered saline (PBS) solution containing various amounts of C17-SAMT (25 to 168 μg), on-line recordings were initiated to observe the effects of the toxin on various parameters, including the excitability threshold and compound muscle action potential (CMAP) amplitude, registered continuously. As shown in Figure 3A, the major effect of C17-SAMT was a dose- and time-dependent decrease of CMAP amplitude. Complete CMAP blockade occurred about 15 min after toxin injection (168 μg/100 μL PBS) to mice.

The dose-response of C17-SAMT effect on CMAPs amplitude revealed an IC_50_ of 50 μg in 30 g of mouse, *i.e.*, 1680 μg/kg (Figure 3C). It is worth noting that the CMAP peak remained perfectly stable before toxin injections (see Figure 2A), or before and 1 h after PBS (100 μL) injections under the same conditions (*i.e.*, 3.64 ± 0.51 *versus* 3.84 ± 0.38, *n* = 4 mice, *p* = 0.41). In addition to the marked CMAP reduction, an increased excitability threshold was also observed (see Figure 2B, middle traces). After C17-SAMT (50 μg/100 μL PBS) injection, the stimulation necessary to produce 50% of maximum CMAP amplitude increased significantly (*p* = 0.02) from 0.35 ± 0.11 to 0.51 ± 0.10 mA (*n* = 4 mice). All these effects were reversible 8–12 h after a given injection (Figure 3B, right traces).

**Figure 4:**
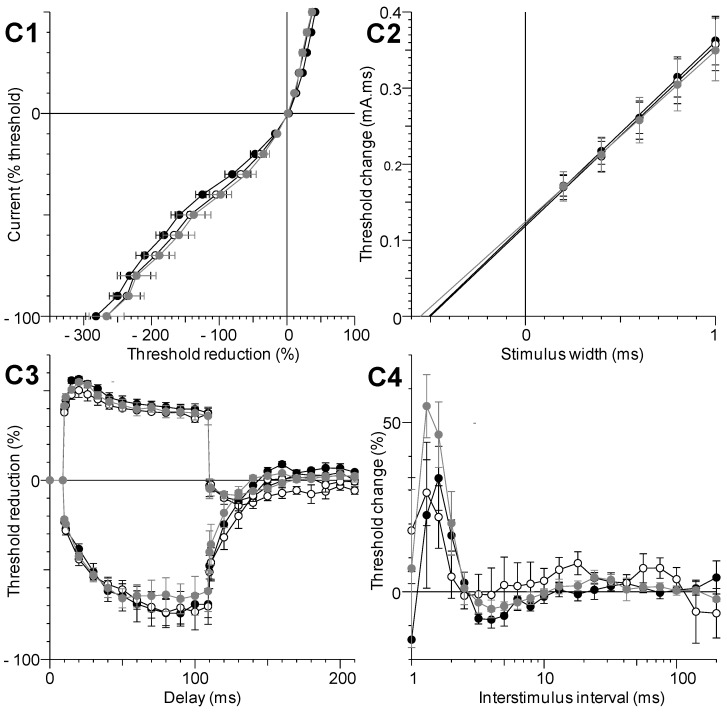
Excitability waveforms recorded, *in vivo*, from tail muscle following caudal motor nerve stimulation in 10 months-old mice before (black circles) and 1 (white circles) and 8 h (grey circles) after injection of 100 μL PBS solution containing 50 μg C17-SAMT. (**C1**) Current-threshold relationships; (**C2**) Charge-duration relationships established from strength-duration testing; (**C3**) Threshold electrotonus; and (**C4**) Recovery cycle. Mean ± SD of data obtained from four different mice.

The five different excitability tests, performed together before and 1 and 8–12 h after C17-SAMT (50 μg/100 μL PBS) injections, did not reveal further effects of the toxin since no apparent modification of excitability waveforms was detected (Figure 4). Furthermore, analyzing the parameters determined from these excitability tests confirmed that no significant difference occurred between these parameters (*p* > 0.13).

Similar results were obtained when recording the tail muscle CMAP before and after injection of 100 μL PBS solution containing various amounts of C17-SPA (from 40 to 280 μg). Indeed, the dose-response of C17-SPA effect on the amplitude of CMAPs revealed an IC_50_ of 108 μg/30 g mouse, *i.e.*, 3600 μg/kg (Figure 3C), a value close to that determined for C17-SAMT.

#### 2.1.6. *In Vitro* Effects of C17-SAMT

**Figure 5:**
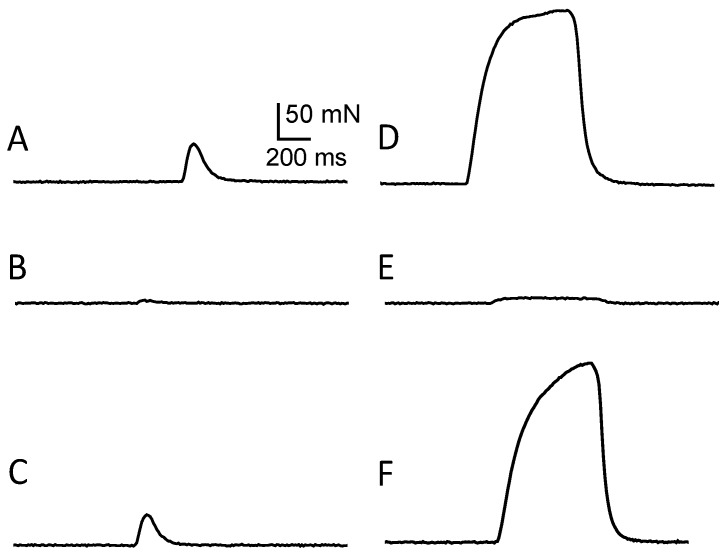
Effect of C17-SAMT on directly-elicited isometric twitch and tetanic contractions of an isolated mouse hemidiaphragm. Representative single twitch (**A**) and tetanic contraction recordings (**D**, 40 Hz) under control conditions, and in the presence of 65 μg/mL C17-SAMT (**B**, single twitch) and (**E**, 40 Hz tetanic contraction). Note the marked block of the twitch and tetanic contractions induced by the toxin (**B**,**E**). (**C**,**F**) Reversal of C17-SAMT effect following 50 min wash out of C17-SAMT from the medium. Vertical calibration in (**A**) applies to all recordings.

C17-SAMT caused a dose- and time-dependent block of directly-elicited isometric contraction of isolated mouse hemidiaphragms. At a low concentration (22 μg/mL), a rather small decrease (20.25% ± 2.18%, *n* = 6) in muscle twitch tension peak amplitude was observed within 7 min. At a higher concentration of C17-SAMT (65 μg/mL), a much more marked decrease occurred, and a blockage of 92.61% ± 1.41% (*n* = 6) was recorded with respect to control (Figure 5A,B). The blockage attained 97.43% ± 1.19% (*n* = 6) following tetanic stimulations, compared to control (Figure 5D,E). Similar results were obtained from indirectly-elicited muscle twitches following both single and tetanic nerve stimulations in mouse phrenic-hemidiaphragm preparations (data not shown).

**Figure 6:**
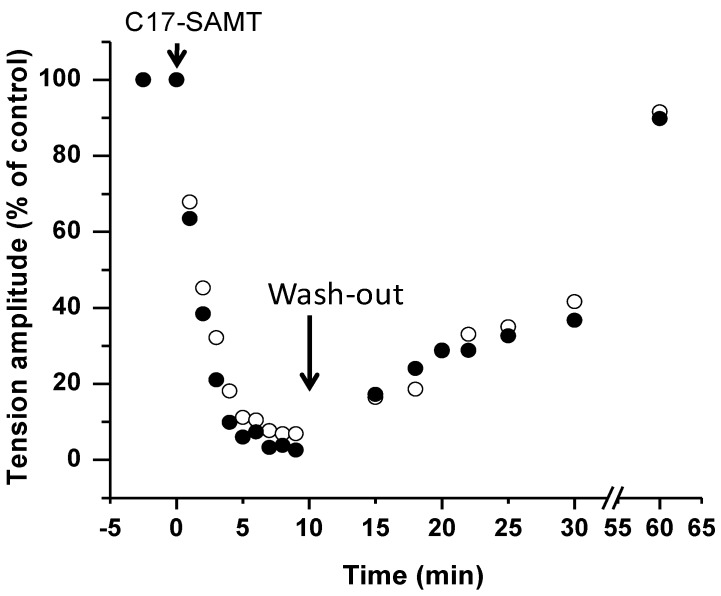
The time-dependent effect of C17 SAMT (65 μg/mL) on twitch (open circles) and tetanic tension amplitude (filled circles) evoked by direct muscle stimulation (in % relative to control). The arrow-head indicates the addition of C17 SAMT to the medium, and the arrow the start of the washout of the toxin from the medium.

The neuromuscular block caused by C17-SAMT was persistent, but could be reversed by a 50 min washout of the toxin from the medium, leading to a 91.6% ± 2.19% (*n* = 3) recovery for directly-elicited muscle twitch, and 89.8% ± 1.89% (*n* = 3) for directly-elicited tetanic contraction (Figure 5C,F and Figure 6).

#### 2.1.7. Causative Organism(s)

**Figure 7:**
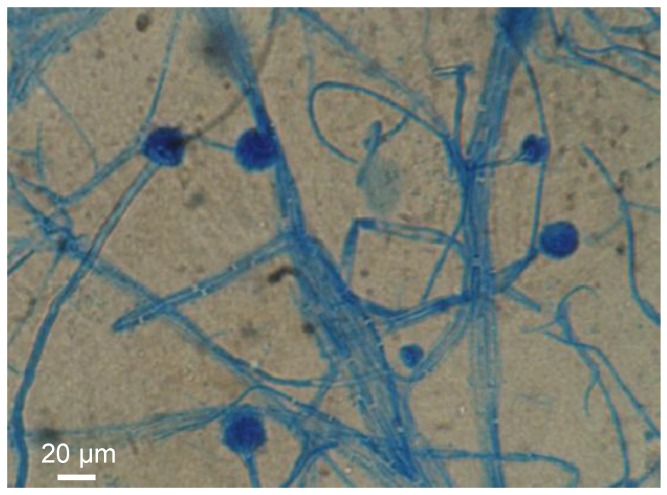
Microscopic observation of *Aspergillus* sp. strain obtained from contaminated mussels.

Both the macroscopic general aspect of cultures and the microscopic aspect (morphological characteristics) of the strains isolated from contaminated mussels were studied. The results revealed the presence of three different strains of marine microfungus, and the genera *Aspergillus* (Figure 7), *Fusarium* and *Trichoderma* were identified. Studies aimed to definitively determine the causative organism(s), *i.e.*, if any of the isolated fungal strains produce toxic compounds and in particular C17-SAMT, are in progress.

### 2.2. Discussion

The detection of marine fungus in toxic samples (*Fusarium*, *Aspergillus* and *Trichoderma* sp.) which are known to produce mycotoxins [12,13], the non-identification of any conventional phycotoxins in contaminated samples, and the evidence from the retention time together with the mass accuracy close to 1 ppm of the toxic compound and C17-SPA shows that the compound responsible for the toxicity of mussels collected from shellfish farming areas in Bizerte Lagoon is unequivocally a mycotoxin structurally similar to C17-SPA. This toxin was thus named C17-sphinganine analog mycotoxin (C17-SAMT).

Recently, 250 marine-derived fungi strains were isolated from mussels (*Mytilus galloprovincialis*) and their immediate environment (marine sediments and seawater) in shellfish farming areas along the Algerian Coast [14]. The major genera detected were * Penicillium*, *Fusarium*, *Aspergillus*, *Trichoderma*, and *Cladosporium*. Data provided from screening studies for extract toxicity clearly confirmed the occurrence of these microorganisms on the Southern Mediterranean shore. It should be noted that these genera were also detected in the North shore in particular in France [4], Greece [15] and Italy [16].

A relationship between shellfish contamination and the presence of marine fungi in the medium have been previously established [17,18,19,20]. Indeed, numerous strains of the genus *Penicillium* have been isolated from shellfish farming areas, and were shown to produce toxic compounds such as griseofulvin [21] or communesins [22]. In the same context, a cytotoxic mycotoxin, named gliotoxin, from *Aspergillus fumigates*, was found in sediments of mussel bed [23].

Sphinganine analog mycotoxins, polyketide-derived natural products, are among the most abundant mycotoxins and, in recent years, many of them were discovered due to advances in LC-MS performance [24].

According to our data, the digestive gland was the most toxic part of mussels, confirming contamination by filtration, and confirming also that microscopic marine fungi found in this study can grow and sporulate in the immediate environment of mussels since this contamination did not affect clams that were collected from coastal sites. It should be noted that biodeposits from suspended mussel cultures on the surrounding surficial sediments were abundant. Suspended culture mussels especially in areas of low hydrological energy and shallow depth, which is the case for Bizerte Lagoon, can generate a favorable environment for the development of many microorganisms such as marine microfungi.

Positive paralytic shellfish poisoning test was due to a slight diffusion in the rest of the meat which explains the toxicity of flesh when the contamination was high. It should be noted that the same contamination processes occurred with phycotoxins [25,26].

*In vivo* recordings of the multimodal excitability properties of mouse neuromuscular system revealed that both C17-SAMT and C17-SPA produce a similar and marked inhibition of CMAPs recorded from the tail muscle, without significant modification of excitability waveforms and corresponding parameters except an increased excitability threshold. This inhibition was completely reversed (within about 8 h) after C17-SAMT (50 μg/100 μL PBS) injections. Furthermore, *in vitro* data on neuromuscular preparations revealed that C17-SAMT blocked both nerve-evoked and directly elicited twitch and tetanus tension responses. Here again, the toxin effects were reversible (within about 1 h) after C17-SAMT wash-out. Several mechanisms may be involved in the inhibition of skeletal muscle contraction such as: (i) Marked membrane depolarization of axons, nerve terminals, and/or skeletal muscle; (ii) Blockade of voltage-gated sodium channels of axons and/or skeletal muscle; and/or (iii) Blockade of muscle-type nicotinic acetylcholine receptors. However, this last possibility seems to be unlikely because blockers of nicotinic receptors usually do not block directly elicited muscle contraction in skeletal muscle [27]. Therefore, it is likely that the C17-SAMT toxicity profile and reversibility of biological activity may be related to the block of an ion channel and most probably to the voltage-gated sodium channels, as shown for other paralytic shellfish toxins like saxitoxin and analogs [28]. Experiments are in progress in order to determine the molecular mechanism of action of the C17-SAMT.

## 3. Experimental Section

### 3.1. Materials

Samples of mussels (*Mytilus galloprovincialis*) were collected weekly between September 2006 and December 2009 from Bizerte Lagoon. Sampling was carried out from shellfish farming areas and controlled by the “Commissariat Régional au Développement Agricole de Bizerte” (CRDA, Bizerte, Northern Tunisia). Samples were kept at 4 °C until analyzed.

Acetonitrile, diethyl ether and dichloromethane were purchased from Panreac Quimica SA (Castellar del Vallès, Barcelona, Spain), acetone from Carlo Erba reagents (Val de Reuil, France), trifluoroacetic acid (TFA) and Tween-60 saline from Sigma-Aldrich (Dublin, Ireland), and a commercial C17-SPA from Avanti Polar Lipids (Alabaster, AL, USA).

### 3.2. Methods

#### 3.2.1. Sample Extraction and Solvent Partition

Sample extraction was performed using methods previously reported [27,29]. Briefly, 20 g of digestive gland from mussels (*Mytilus galloprovincialis*) were minced and extracted three times with 50 mL acetone, each time using a screw mixer. The combined acetone extract was filtered and evaporated to dryness in a rotary evaporator with a temperature-controlled bath. The residual aqueous layer was defatted with diethyl ether (1:1) and extracted with dichloromethane (1:1) three times. Dichloromethane, diethyl ether and aqueous layers were evaporated to dryness and suspended in 1 mL stock solution of MilliQ water to be used for toxicity assays and chromatographic analysis.

#### 3.2.2. Toxicity Assays

Toxicity analyses were carried out using the mouse bioassay adapted from that previously described [30]. Each of the mussel extracts or purified toxic compound was diluted in 1% Tween-60 saline and injected i.p. into male adult Swiss-Webster mice (20 g, two-four groups of three mice receiving 1 mL/mouse in each case). Control mice (male adult Swiss-Webster mice of 20 g weight) were injected i.p. with only 1% Tween-60 saline (two groups of three mice receiving 1 mL/mouse). The mice were observed up to 24 h for signs of illness, and death times were recorded. Increasing amounts of purified toxic compound were also orally administered to mice, directly into their lower oesophagus, in 0.2 mL of MilliQ water. Male C57BL/6 mice (weighing 20 g) were also used for i.c.v. injections of increasing amounts of mussel extracts or purified toxic compound diluted in 0.9% (w/v) NaCl (two-four groups of three mice receiving 5 μL/mouse in each case). The mice behavior and survival time were observed for up to 24 h.

#### 3.2.3. Chromatographic Analysis

Following liquid/liquid extraction, the water-soluble extract, which possessed the entire toxic activity, was analyzed by reversed-phase HPLC using a C18 symmetry column (4.6 × 250 mm, 5 μm; Waters SAS, Guyancourt, France). Elution was done with a mobile phase composed of a gradient of solvent A (aqueous phase: Milli-Q water + TFA (0.1%)), and solvent B (organic phase: acetonitrile + TFA (0.1%)), whose proportions were controlled by a programmable pump system (Agilent 1100 series, Agilent Technologies, Santa Clara, CA, USA). A linear gradient from 20% to 60% B was run between 2 and 35 min. The column effluent was monitored at 210 and 280 nm with a UV absorbance detector (Agilent 1100 series). Temperature was fixed to 25 °C, and the run lasted for 20 min. Series of fractions were hand-collected, lyophilized, and tested for activity. The active fraction was further purified using a C18 GOLD ODS column (4.6 × 150 mm, 5 μm; Thermo Fisher Scientific, Bremen, Germany) under the same conditions, as reported above. Individual fractions were collected, lyophilized, and stored at −20 °C until use.

#### 3.2.4. HPLC-ESI-LC Analysis

The HPLC equipment was formed by a Dionex UltiMate^®^ 3000 binary system (Thermo Fisher Scientific, Bremen, Germany). Samples were carried out with an analytical manual injection valve for UltiMate^®^ 3000 1G Pump Series with 25 μL sample loop (Thermo Fisher Scientific). The column used was a Zorbax SB-C18 of 1 mm diameter, 15 mm length and 3 μm granulometry. The products were eluted using a linear gradient between 15% and 80% of acetonitrile in 40 min and then increased to 100% in 5 min. This system was coupled to a mass spectrometer LTQ-ORBITRAP instrument (Thermo Fisher Scientific) equipped with an electrospray (ESI) source. The injection volume was 25 μL. The mobile phase for analysis was solvent A (water 0.1% formic acid) and solvent B (acetonitrile 0.09% formic acid). Mass measurement was done in positive mode using the orbitrap set to a resolution of 60,000 at *m*/*z* 400. The automatic gain control was fixed to a target of 5 × 10^6^. The scan was set between *m*/*z* 200 and 1700. Data were analyzed using Thermo Scientific Xcalibur software.

#### 3.2.5. Determination of Toxin Concentration by LC-MSD-Trap-XCT

Toxin concentration was estimated using LC/MSD Trap XCT instrument (Agilent 1100 series) equipped with a C18 column (4.6 × 25 cm, 5 μm, Eurospher, KNAUER, Berlin, Germany). Certified solution of C17-SPA (5 mg/mL) was used to calibrate, and peak areas were measured to express peak intensities.

#### 3.2.6. *In Vivo* Study on the Mouse Neuromuscular System

The multimodal excitability properties of the mouse neuromuscular system (Swiss females at 10.4 ± 1.8 weeks of age and weighting 30.0 ± 1.6 g (*n* = 30)) were assessed, *in vivo*, by means of minimally-invasive electrophysiological methods, using the Qtrac^©^ software written by Hugh Bostock (Institute of Neurology, London, UK), as previously described [31]. The experiments were performed in accordance with the guidelines established by the French Council on animal care “Guide for the Care and Use of Laboratory Animals”: EEC86/609 Council Directive—Decree 2001-131, on mice under anesthesia, by means of isoflurane (AErrane^®^, Baxter S.A., Lessines, Belgium) inhalation, and the experimental protocols were approved by the French Departmental Direction of Animal Protection (Number A91-453 to Evelyne Benoit).

Briefly, the electrical stimulations were delivered to the caudal motor nerve (at the base of the tail), by means of surface electrodes, and CMAPs were recorded using needle electrodes inserted into the tail muscle. To study the underlying mode of action of C17-SAMT, compared to C17-SPA, intramuscular (i.m.) injections of PBS solution containing various amounts of C17-SAMT (from 8.5 to 168 μg) or C17-SPA (from 40 to 280 μg) were delivered at the base of mouse tail, between stimulation and ground electrodes. Immediately after a given injection, on-line recordings were initiated to observe the effects of C17-SAMT or C17-SPA on some selected excitability parameters, such as the excitability threshold and CMAP amplitude, registered continuously. To identify the duration of effect(s) of C17-SAMT or C17-SPA, five different excitability tests (stimulus-response, strength-duration and current-threshold relationships, as well as threshold electrotonus and recovery cycle; [32]) were performed together before and 1–12 h after a given injection. As a whole, more than thirty parameters were determined from the five different excitability tests, and analyzed. It is worth noting that each specific excitability test provides additional and complementary information regarding the functional status of ion channels and electrogenic pumps, as well as membrane properties of the neuromuscular system [33,34,35].

Data are expressed as means ± SD, and differences between values were tested using the parametric unpaired two-tailed *t*-test, two-way ANOVA, or the non-parametric Mann-Whitney *U*-test, depending on the equality of variances estimated using the Lilliefors test. Differences were considered significant when *p* < 0.05.

#### 3.2.7. Twitch Tension Recordings on Isolated Mouse Neuromuscular Preparations

For isometric twitch tension measurements, hemidiaphragms with their respective associated phrenic nerves, or extensor digitorum longus (EDL) muscles were carefully isolated from Swiss-Webster mice (20–25 g) killed by dislocation of cervical vertebrae followed by immediate exsanguination. Isolated neuromuscular preparations were mounted in silicone-lined organ baths (4 mL volume) and superfused with a standard Krebs-Ringer solution of the following composition (in mM): 154 NaCl, 5 KCl, 2 CaCl_2_, 1 MgCl_2_, 5 *N*-2-hydroxyethylpiperazine-*N*′-2-ethane-sulphonic acid (Hepes) buffer and 11 glucose. The solution, gassed with pure O_2_, had a pH value of 7.4. The phrenic nerve of isolated hemidiaphragms was stimulated with a suction microelectrode, adapted to the diameter of the nerve, with pulses of 0.1 ms duration and supramaximal voltage (typically 3–8 V) supplied by an S-44 stimulator (Grass Instruments, West Warwick, RI, USA) at either 0.1 or 40 Hz. For direct muscle stimulation, an electrode assembly was placed along the length of the fibers, and 20 μM d-tubocurarine (Sigma-Aldrich, Saint Quentin Fallavier, France) added to the medium to block neuromuscular transmission. For each preparation investigated, the resting tension was adjusted to obtain maximal contractile responses upon indirect and direct muscle stimulations. Tension signals from the isometric transducer were amplified, collected and digitized as previously described [36]. Computerized data acquisition and analysis were performed with the WinWCP V3.9.6 software program kindly provided by John Dempster (University of Strathclyde, Glasgow, Scotland, UK).

Data are presented as their mean ± SEM. Comparison was made using Student’s *t*-test. A difference was considered to be significant when *p* < 0.05.

#### 3.2.8. Fungal Culture and Strain Identification

Mussels were opened after careful decontamination of the shell. The meat was washed with sterile distilled water and mixed. After centrifugation at 2500 rpm for 10 min, the supernatant was seeded onto agar (1 mL per dish). Fungal cultures were performed in Sabouraud medium and then poured into 20 cm diameter Petri dishes. For each sample, two dishes were incubated at different temperatures (12 and 27 °C). Fungal strains were isolated over a period of four months (which corresponds to the peak of toxicity). They were identified only by genus, according to the Pitt’s method based on the determination of microscopic characters and colony aspect of cultured isolated strains [37].

## 4. Conclusions

This is the first report of contamination of mussels from Tunisia by marine fungal toxins. C17-SAMT was identified as the toxic compound responsible for episodes of toxicity found in Bizerte Lagoon since the summer of 2006. *In vivo* and *in vitro* studies on the mouse neuromuscular system demonstrated that this mycotoxin exerts its effects by blocking skeletal muscle contraction, which can explain some of the symptoms observed during acute toxicity assays. Further studies must be conducted to determine the molecular mechanism of action of this toxin and its eventual toxicological effects to wild animals and humans.
